# Tobacco Smoke

**Published:** 1894-08

**Authors:** 


TOBACCO SMOKE.—There is a popular notion among smokers that tobacco smoke acts as an antiseptic. This whim, however, is not altogether supported by scientific investigation, and, until very recently, the results of exposing the bacilli of various diseases to tobacco smoke were not known. The investigations and experiments of Professor Vincenzo Tassinari, of the University of Pisa, Italy, have now proved that tobacco smoke is, to a certain extent, an annihilator of disease by its action upon the growth of bacilli. Dr. Tassinari has taken great pains to demonstrate its utility in that direction, and has constructed special apparatus for the purpose. In order to imitate as closely as possible the process going on in the human mouth during the inhalation of smoke, Dr. Tassinari passed tobacco smoke through a horizontal tube into a chamber kept moist by a bunch of wet cotton batting suspended in it, and containing in addition, some carefully nurtured bacilli, which were submitted to the action of the smoke. The professor used, in his experiments, the various qualities of tobacco usually smoked in Italy —three kinds of cigars, and the best Turkish cigarette tobacco. The action of all these was tried upon seven classes of microbes, including those of typhus fever, pleuro-pnuemonia, cattle distemper, and the bacillus which is believed to be the cause of cholera. The results were very remarkable, and amply repaid Dr. Tassinari for his labor; the experiments showed unmistakably that tobacco smoke retards the growth of some varieties of bacteria, while it prevents the development of others.

Dr. Tassinari carried his investigations further, actually fixing the length of time during which the development of the bacilli is prevented. By comparing experimentally the growth of the same micro-organisms when not exposed to the retarding action of smoke, and their development during the time of such exposure, it was found that the smoke obtained from large "Cavour" cigars, for example, delayed the development of cattle distemper bacilli for one hundred hours, and that the same smoke absolutely prevented the formation of cholera and typhus bacteria—in fact, acted as a germicide. Similar results were obtained in the experiments with other descriptions of manufactured tobacco.

Dr. Tassinari attributes the annihilating effect of tobacco smoke upon bacteria to the action of the chemical elements contained in it. He is continuing his experiments, with rgard especially to the action of tobacco smoke upon bacillus of tuberculosis, and if these should prove as conclusive as those he has made with the microorganisms mentioned above, it is not improbable, that tobacco smoke may be utilized in the treatment of consumptive patients.

The highly important results of Professor Tassinari's observations should, and will receive careful attention from physicians and scientists.—*Doctor and Druggist.*

